# Conductive polythiophene/graphitic-carbon nitride nanocomposite for the detection of ethanol mixing in petrol

**DOI:** 10.1039/d3ra00381g

**Published:** 2023-04-18

**Authors:** Ahmad Husain, Sharique Ahmad, Sara A. Alqarni, Samar J. Almehmadi, Mudasir A. Yatoo, Faiza Habib, Mohd Urooj Shariq, Mujahid Ali khan

**Affiliations:** a Department of Mechanical Engineering, Indian Institute of Technology Ropar Punjab 140001 India ahmadhusain2065@gmail.com shariqueahmad14@gmail.com; b Applied Science and Humanities Section, University Polytechnic, Faculty of Engineering and Technology, Aligarh Muslim University Aligarh 202002 India; c Department of Chemistry, College of Science, University of Jeddah Jeddah Saudi Arabia; d Department of Chemistry, Faculty of Applied Science, Umm-Al-Qura University Makkah-24230 Saudi Arabia; e Department of Materials, Faculty of Engineering, Imperial College London SW7 2AZ UK; f Department of Chemistry, University College London WC1H 0AJ UK; g Department of Chemistry, Faculty of Science, Aligarh Muslim University Aligarh 202002 India

## Abstract

The automobile vehicles must be operated on fuel containing no more than 10% ethanol. Use of fuel having more than 10% ethanol may cause engine malfunction, starting and running issues, and material degradation. These negative impacts could cause irreversible damage to the vehicles. Therefore, ethanol mixing in petrol should be controlled below 10% level. The current work is the first to report sensing of ethanol mixing in petrol with reference to the variation in the DC electrical conductivity of polythiophene/graphitic-carbon nitride (PTh/gC_3_N_4_) nanocomposite. The *in situ* chemical oxidative method of polymerization was used for synthesizing PTh and PTh/gC_3_N_4_ nanocomposite. Fourier transform infrared spectroscopy (FT-IR), X-rays diffraction (XRD), thermo-gravimetric analysis (TGA), transmittance electron microscopy (TEM) as well as scanning electron microscopy (SEM) analysis were used for confirmation of the structure along with morphology of the PTh and PTh/gC_3_N_4_ nanocomposite. The thermal stability of DC electrical conductivity of PTh and PTh/gC_3_N_4_ nanocomposite were tested under isothermal and cyclic ageing condition. The sensing response of PTh and PTh/gC_3_N_4_ nanocomposite as a function of DC electrical conductivity were recorded in petrol and ethanol atmosphere. The sensing response of PTh/g-C_3_N_4_ nanocomposite in petrol atmosphere was 6.1 times higher than that of PTh with lower detection limit to 0.005 v/v% of ethanol prepared in *n*-hexane.

## Abstract 2

Conductive polythiophene/graphitic-carbon nitride nanocomposite for the detection of ethanol mixing in petrol.

## Graphical abstract

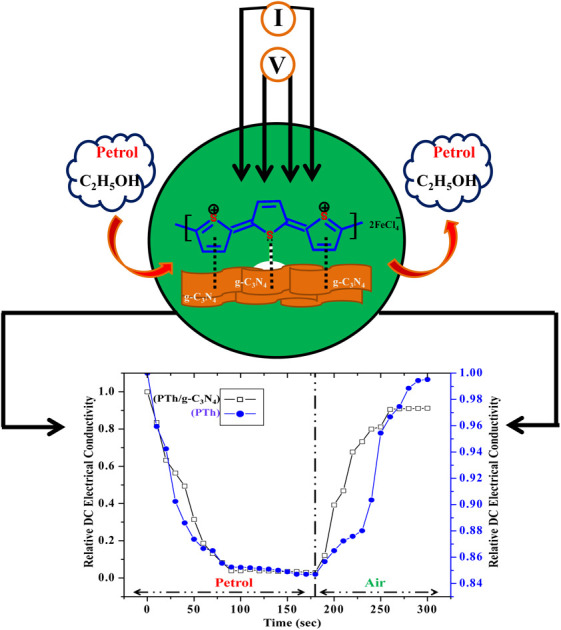

## 1. Introduction

Ethanol, as a fuel produced mostly from plants like sugarcane and maize, is a desirable alternative to gasoline for reducing reliance on fossil fuels and lowering CO_2_ net emissions into the environment. Furthermore, ethanol has a greater octane rating than gasoline,^1^ indicating that ethanol–gasoline blends have a higher octane rating than regular gasoline. However, according to a recent analysis, ethanol–gasoline blends increase vehicle emissions of volatile organic compounds (VOCs), predominantly acetaldehydes compounds, which act as precursors to tropospheric ozone in urban areas.^2^ These outcomes have caused authorities to reconsider their previous support for the use of ethanol–gasoline mixes in major cities, where a high tropospheric ozone concentration is the primary environmental concern. Use of fuel containing more than 10% ethanol may cause materials degradation, starting and operating issues, and automobile engine malfunction. Automobiles can sustain irreparable damage as a result of these negative effects. Ethanol increases the danger of groundwater along with soil pollution, owing to a rise in tank corrosion, decreasing the interfacial tension between NAPL–water and lastly by preventing biodegradation and increasing the contaminant solubility. Besides its adverse effect, ethanol blending in petrol is used extensively all over of the world therefore selectively and rapidly detection to monitoring the percentage of ethanol in petrol is highly needed.^3,4^

Many prospective materials have been studied for their behavior in chemical sensing throughout the years, as well as the sensing mechanisms that underlie the unique interactions of vapours, chemicals as well as gases with materials that are used in sensing applications. The development of chemical/gas/vapour sensors has recently focused heavily on carbon nanomaterials, metal oxide semiconductors, conductive polymers (CPs) and their nanocomposites.^5–18^ Due to its extraordinary functionalities through regulated charge transfer process, adjustable electrical conductivities, and self-resistivity changes upon exposure to various gases, conducting polymers have long been sought after as chemiresistor materials.^16–21^ Due to its extraordinary functionalities through regulated charge transfer process, adjustable electrical conductivities along with self-resistivity which alters when exposed to variety of gases, CPs have for a long time been used as chemiresistor.^9–13^ Polythiophene (PTh) being chemically, thermally and environmentally highly durable is amongst the most researched CPs. PTh and its derivatives stood out among gas sensors owing to their distinctive doping and de-doping mechanisms that produce a change in electrical conductivity when exposed to various chemicals and gases.^22–33^

2-Dimensional nanomaterials have captured a significant deal of attention due to its several uses in the past ten years and their extremely high surface area to volume ratio. Researchers have been fascinated by MXene, graphene, g-C_3_N_4_, and MoS_2_ among these materials because of their remarkable capacity as sensing materials, supercapacitors, and hydrogen evolution reactions.^34–46^ Taking inspiration from the aforementioned properties, here we described our efforts in developing polythiophene (PTh) and polythiophene/graphitic-carbon nitride (PTh/g-C_3_N_4_) nanocomposites for ethanol sensing in petrol. This is, too are awareness, the principal attempt which employs a sensor based on PTh/g-C_3_N_4_ nanocomposite for detecting ethanol in petrol at ambient conditions. We have also examined the sensing performance of PTh/g-C_3_N_4_ nanocomposite at different v/v% of ethanol in *n*-hexane.

## 2. Experimental

### 2.1. Materials

Thiophene (E. Merck), melamine (Fisher Scientific), chloroform (Fisher Scientific), anhydrous ferric chloride (CDH), methanol (E. Merck) and acetone (E. Merck) were used in their original state. Double distilled water (DDW) was used throughout the experiments.

### 2.2. Preparation of graphitic-carbon nitride (g-C_3_N_4_)

The light-yellow g-C_3_N_4_ powder was made by heating melamine with heating rate 15 °C min^−1^ upto 550 °C for 3.5 h in an alumina crucible. Then the vessel containing g-C_3_N_4_ powder was cooled to room temperature for further processing.^47^

### 2.3. Preparation of polythiophene (PTh) and polythiophene/graphitic-carbon nitride (PTh/g-C_3_N_4_) nanocomposite

PTh and PTh/g-C_3_N_4_ nanocomposites were prepared by the *in situ* chemical oxidative polymerization. The first step was dissolving thiophene (2 mL) in chloroform (40 mL), followed by ultrasonication for 25 minutes. Additionally, another solution of a known amount of g-C_3_N_4_ (20%) was prepared by adding it to chloroform (60 mL) and ultrasonically sonicated for 30 minutes. The resulting solution containing the g-C_3_N_4_ sheets was then poured in the thiophene solution and this mixture was then ultrasonicated for 110 minutes. The thiophene monomers got absorbed on the g-C_3_N_4_ sheets during ultrasonication. Following this, a separate solution of 16.24 g (100 mmol) ferric chloride in chloroform (100 mL) was made by stirring for 20 minutes to obtain a homogeneous mixture. Now, for polymerizing thiophene monomers on the surface of g-C_3_N_4_ sheets, suspension of FeCl_3_ was added dropwise in thiophene and g-C_3_N_4_ mixture followed by constant stirring on magnetic stirrer. After 24 h of stirring, the as-prepared PTh/g-C_3_N_4_ nanocomposite was collected by Buchner suction funnel after draining of the residual in the solution and then washed numerous times using methanol, subsequently by double distilled water and lastly by acetone. Amid washing with methanol, the colour of the material changed dark brown from deep black. The nanocomposites were lastly dried in a vacuum oven at 60 °C for 24 hours. Finally, using a mortar and pestle, these items were ground into a fine powder. An analogous procedure was used to produce the pure PTh nanoparticles.

### 2.4. Morphological and structural characterization

The characterization of PTh and PTh/g-C_3_N_4_ nanocomposites was performed by FT-IR (PerkinElmer 1725 instrument on KBr pellets), XRD (Bruker D8 diffractometer with Cu Kα radiation at 1.5418 Å), TGA {PerkinElmer (Pyris Diamond) instrument}, SEM {JEOL, JSM, 6510-LV (Japan)}, and TEM {JEM 2100, JEOL (Japan)} techniques respectively.^21–25,30–33^

DC electrical conductivities and sensing experiments were performed by four-in-line probe instrument attached with the PID controlled oven manufactured by Scientific Equipment, Roorkee, India. The thermal stability as a function of DC electrical conductivity under isothermal and cyclic ageing environments was evaluated for all the nanocomposites. The equation which was utilized for calculating the conductivity was as follows:

1 *σ* = [ln 2(2*S*/*W*)]/[2π*S*(*V*/*I*]

where: *V*, *S*, *W* and *I* represent the voltage (V), probe spacing (cm), the pellet-thickness (cm), current (A) and *σ* represents the DC electrical conductivity (S cm^−1^) respectively.^22,23^ 250 mg of each sample was pelletised at room temperature using a hydraulic pressure instrument which operated at a pressure of 70 kN for 3 min.^21–25,30–33^

## 3. Results and discussion

### 3.1. FT-IR

**Fig. 1:**
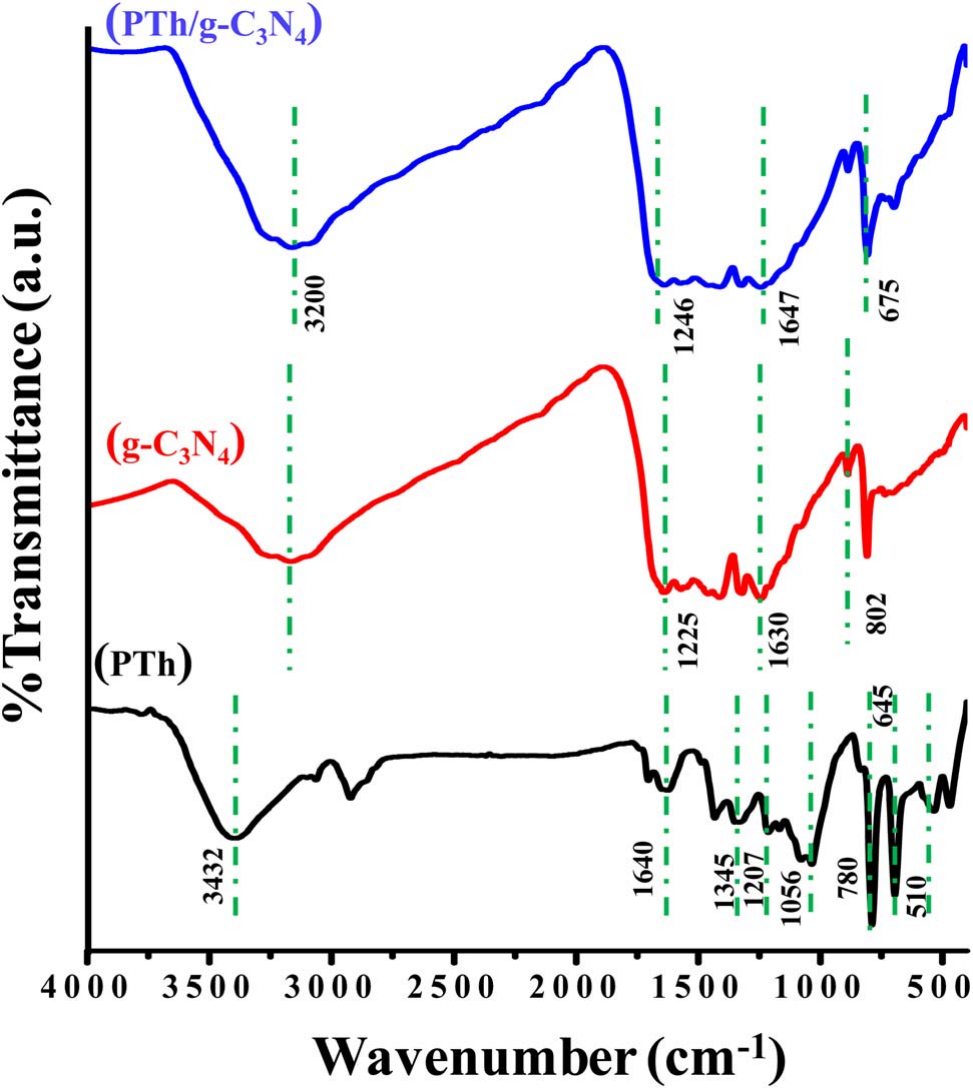
FT-IR spectra of PTh, g-C_3_N_4_, and PTh/g-C_3_N_4_ nanocomposite.

The FT-IR spectra of PTh, g-C_3_N_4_, and PTh/g-C_3_N_4_ are presented in Fig. 1. In the spectra of PTh, the wide peak at 3432 cm^−1^ may appear as a result of O–H stretching frequency of water molecules. The peaks at 1640 cm^−1^ and 1345 cm^−1^ could possibly be due to the C=C, and C–C stretching mode of vibration in the polythiophene rings respectively. The peaks at 1207 cm^−1^ and 1056 cm^−1^ may be because of the bending vibration in PTh. The peak around 780 cm^−1^ reveals the out of plane C–H bond bending in polythiophene. The peak at 645 cm^−1^ is because of the bending mode of vibration of the C–S bond of the thiophene ring. The C–S–C ring deformation mode is the cause of the band at 510 cm^−1^.^22–25^ In the g-C_3_N_4_ spectrum, multiple pinnacles between 1225–1630 cm^−1^ may be due to symmetrical C–N stretching vibration and the pinnacle at 802 cm^−1^ may be traced as dissymmetrical mode of stretching of C–N and C=N as well triazine units of the aromatic rings.^48,49^ In PTh/g-C_3_N_4_, it is found that on mixing of PTh in g-C_3_N_4_, the shoulder shifted to 1246 to 1647 cm^−1^, affirming that the thiophene monomer gets interacted with g-C_3_N_4_. The water molecules which may be trapped by the PTh/g-C_3_N_4_ nanocomposite and the amine groups present on g-C_3_N_4_ showing a merged peak at around 3200 cm^−1^. The peak seen at 675 cm^−1^ may be because of the C–S bending vibrations while all other characteristics peaks of polythiophene get merged with the g-C_3_N_4_.

### 3.2. X-Ray diffraction studies

**Fig. 2:**
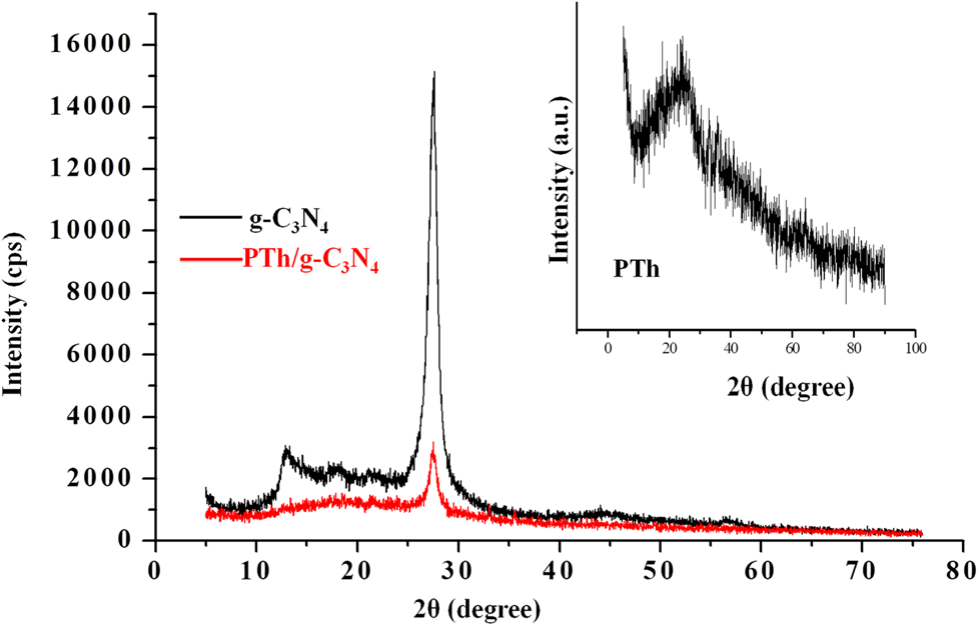
XRD spectra of and PTh, g-C_3_N_4_ and PTh/g-C_3_N_4_ nanocomposite.

Fig. 2 depicts XRD patterns of g-C_3_N_4,_ PTh and PTh/g-C_3_N_4_ nanocomposite. The appeared peaks at 2*θ* = 27.25° and 2*θ* = 12.87° in g-C_3_N_4_ spectra are in accordance with the (002) and (001) Brag reflections, originated from the 2D structure of g-C_3_N_4_.^48,49^ In PTh spectra a wide hump is observed at about 2*θ* = 16–27°, indicating an amorphous form of PTh.^22–25^ In case of PTh/g-C_3_N_4_ nanocomposite a broad bump around 2*θ* = 15–25° and a sharp peak at 2*θ* = 27.48° are observed, illustrating that PTh and g-C_3_N_4_ are present in the nanocomposite. The low intensities peaks in PTh/g-C_3_N_4_ nanocomposite clarifying that the amorphous PTh matrix might have shadowed the high intensity peaks of g-C_3_N_4_, which could be further understood by SEM and TEM studies of PTh/g-C_3_N_4_ nanocomposite. The shift in the diffraction peak, suggested that an interaction existed between g-C_3_N_4_ and PTh in PTh/g-C_3_N_4_ nanocomposite.

### 3.3. Scanning electron microscopy

**Fig. 3:**
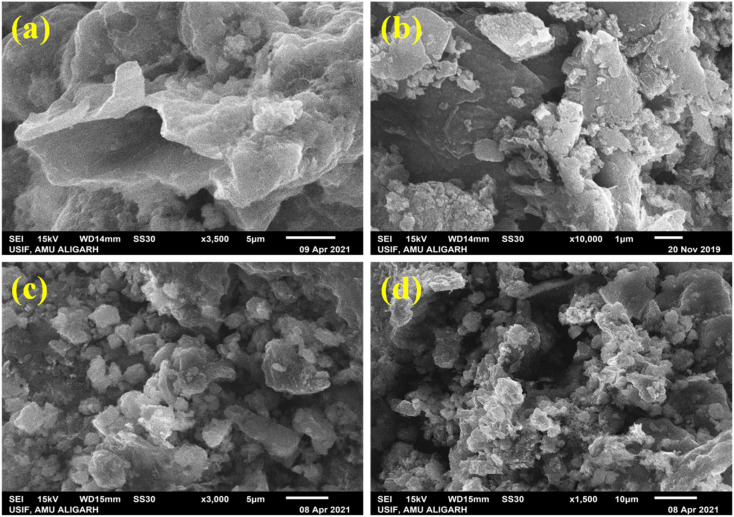
SEM images of: (a) PTh, (b) g-C_3_N_4_, and {(c) and (d)} PTh/g-C_3_N_4_ nanocomposite.

The morphologies of PTh {Fig. 3(a)}, g-C_3_N_4_ {Fig. 3(b)} and PTh/g-C_3_N_4_ {Fig. 3(c and d)} are depicted in Fig. 3 at different magnification by SEM operated at 15 kV. The SEM image of PTh is found to be some flaky and cloudy as shown in Fig. 3(a), while the g-C_3_N_4_ appeared in some sheet like form as shown in Fig. 3(b). The change in morphology of the PTh/g-C_3_N_4_ nanocomposite is evident from Fig. 3(c and d), which seems entirely unlike pure PTh due to the excellent multilayer enveloping of PTh over g-C_3_N_4_ nanosheets. For the PTh/g-C_3_N_4_ nanocomposite, PTh is well mounted on g-C_3_N_4_ nanosheets, resulting in the morphology becoming flaky and sheet-like. Absence of free g-C_3_N_4_ nanosheets are visible in the PTh/g-C_3_N_4_ nanocomposite, it is possible to conclude that PTh effectively covers g-C_3_N_4_ nanosheets.

### 3.4. Transmission electron microscopy

**Fig. 4:**
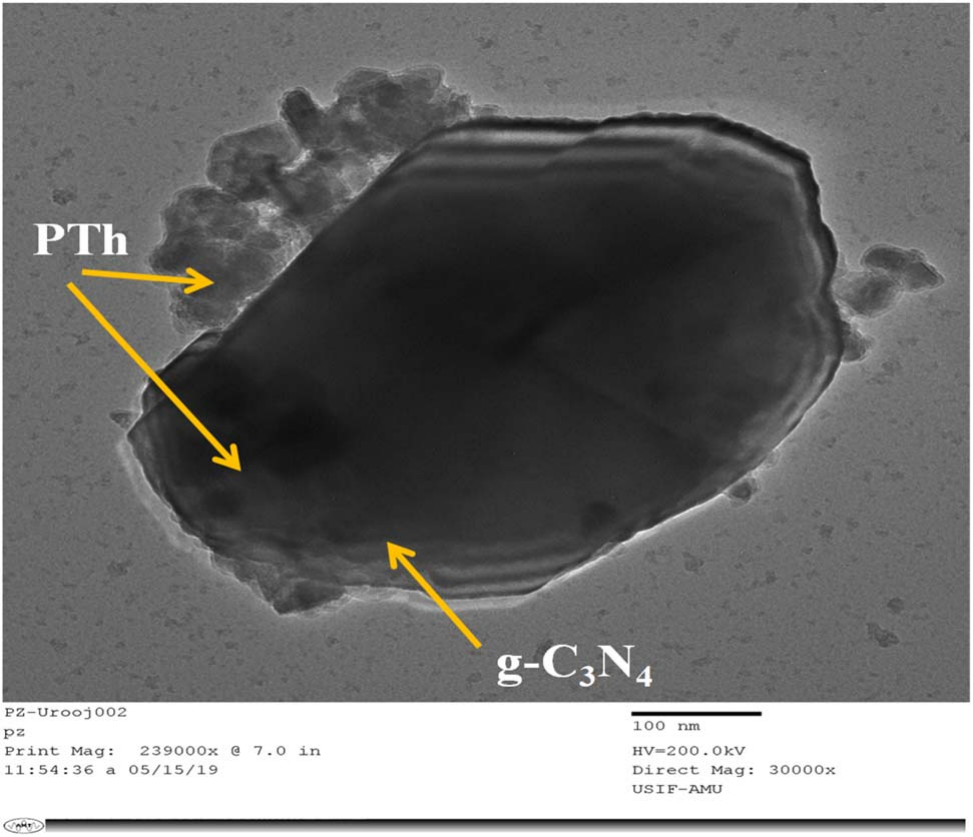
TEM image of PTh/g-C_3_N_4_ nanocomposite.

Fig. 4 displays the TEM micrograph of PTh/g-C_3_N_4_. Here in this image, it can be clearly observed that there is sheet of g-C_3_N_4_ upon which PTh has been successfully deposited and masked upon the entire surface of the g-C_3_N_4_ sheet. The monomers of thiophene polymerize over the large surface of g-C_3_N_4_ providing an effective π-conjugated system which promotes excessive transit of the charge carriers, which the reason for PTh/g-C_3_N_4_ showing higher sensing performance towards ethanol.

### 3.5. Thermogravimetric analysis

**Fig. 5:**
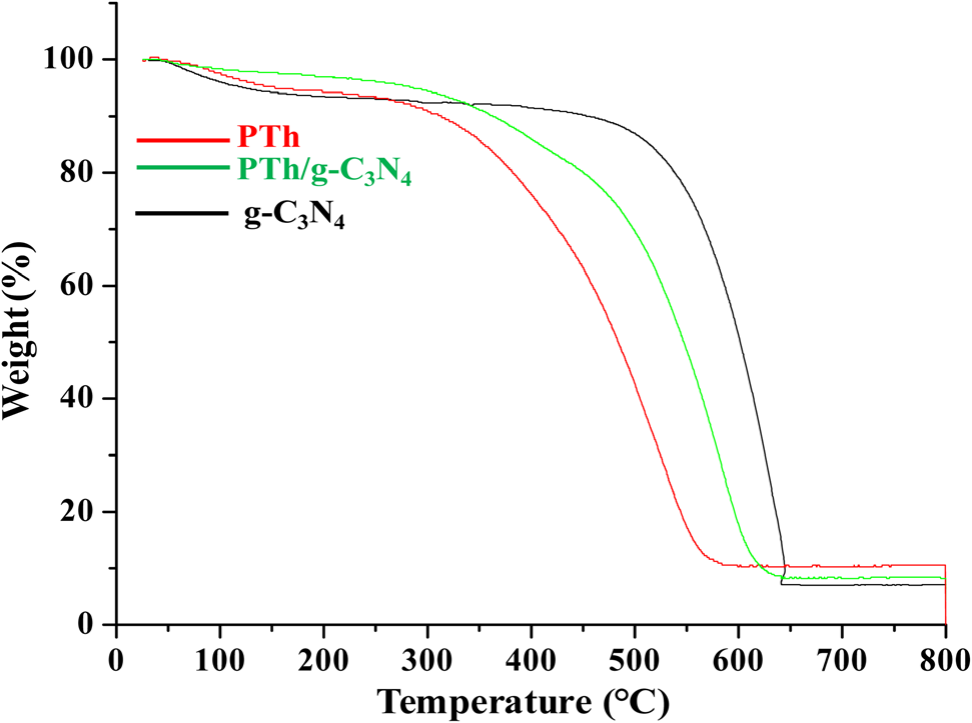
Thermogravimetric analysis of PTh, g-C_3_N_4_ and PTh/g-C_3_N_4_ nanocomposite.

Thermo-gravimetric analysis of PTh, g-C_3_N_4_ and PTh/g-C_3_N_4_ nanocomposite is depicted in Fig. 5. In the degradation procedure of g-C_3_N_4_, the initial weight loss was found from 50 to 120 °C due to loss of water molecules. Subsequently, the oxidative thermal degradation of g-C_3_N_4_ is started at around 510 °C. While the PTh found to be least thermally stable among all of the samples. The initial loss of weight in PTh may be as a result of vaporization of water molecules followed by further degradation started around at 275 °C. In case of PTh/g-C_3_N_4_ nanocomposite, after losing of water molecules at around 80 to 105 °C, the final degradation started from 350 °C which is higher than that of PTh but lower than g-C_3_N_4_, suggesting the higher thermal stability of PTh/g-C_3_N_4_ nanocomposite than that of PTh, which may be a result of the electronic interaction taking place amid PTh and g-C_3_N_4_ nanosheets.

## 4. Electrical conductivity studies

**Fig. 6:**
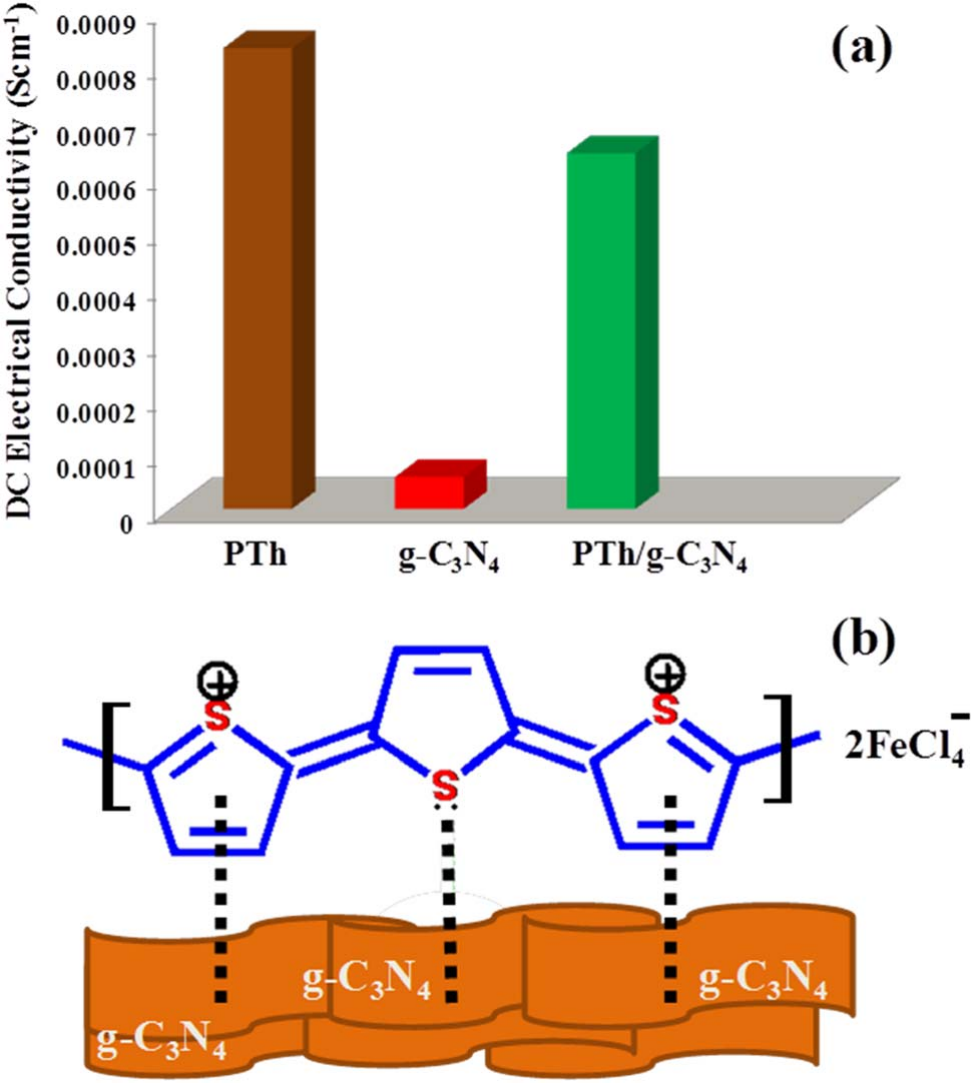
(a) Initial DC electrical conductivity of PTh, g-C_3_N_4_ and PTh/g-C_3_N_4_ nanocomposite and (b) the possible interaction between PTh and g-C_3_N_4_ sheets in PTh/g-C_3_N_4_ nanocomposite.

Fig. 6 the DC electrical conductivities of PTh, g-C_3_N_4_ and PTh/g-C_3_N_4_ nanocomposite were calculated by a 4-in-line probe method and marked as 8.326 × 10^−4^ S cm^−1^, 5.8273 × 10^−5^ S cm^−1^ and 6.4327 × 10^−4^ S cm^−1^ respectively as shown in Fig. 6(a). It was anticipated that upon interacting of lone pairs of S-atoms and π-electrons of thiophene ring in polythiophene with g-C_3_N_4_ sheets, the electrical conductivity will increase by generating more polarons on PTh as depicted in Fig. 6(b). Though, it is expected that there was a decline in electrical conductivity upon g-C_3_N_4_ in PTh may be due the insulating nature of g-C_3_N_4_. However, the fall in DC electrical conductivity was not as much and it is still found in the range of PTh, might be due to the more dominating effect of generated polarons in PTh than insulating g-C_3_N_4_ in tug of war between them. After all, despite an insignificant loss of electrical conductivity in PTh/g-C_3_N_4_ nanocomposite on incorporation of g-C_3_N_4_ in PTh, it provides a high surface area in nanocomposite by the formation of effective arrangement of the π-conjugated system of PTh on the large g-C_3_N_4_ surface area.

### 4.1. Retention of DC electrical conductivity under isothermal ageing condition

**Fig. 7:**
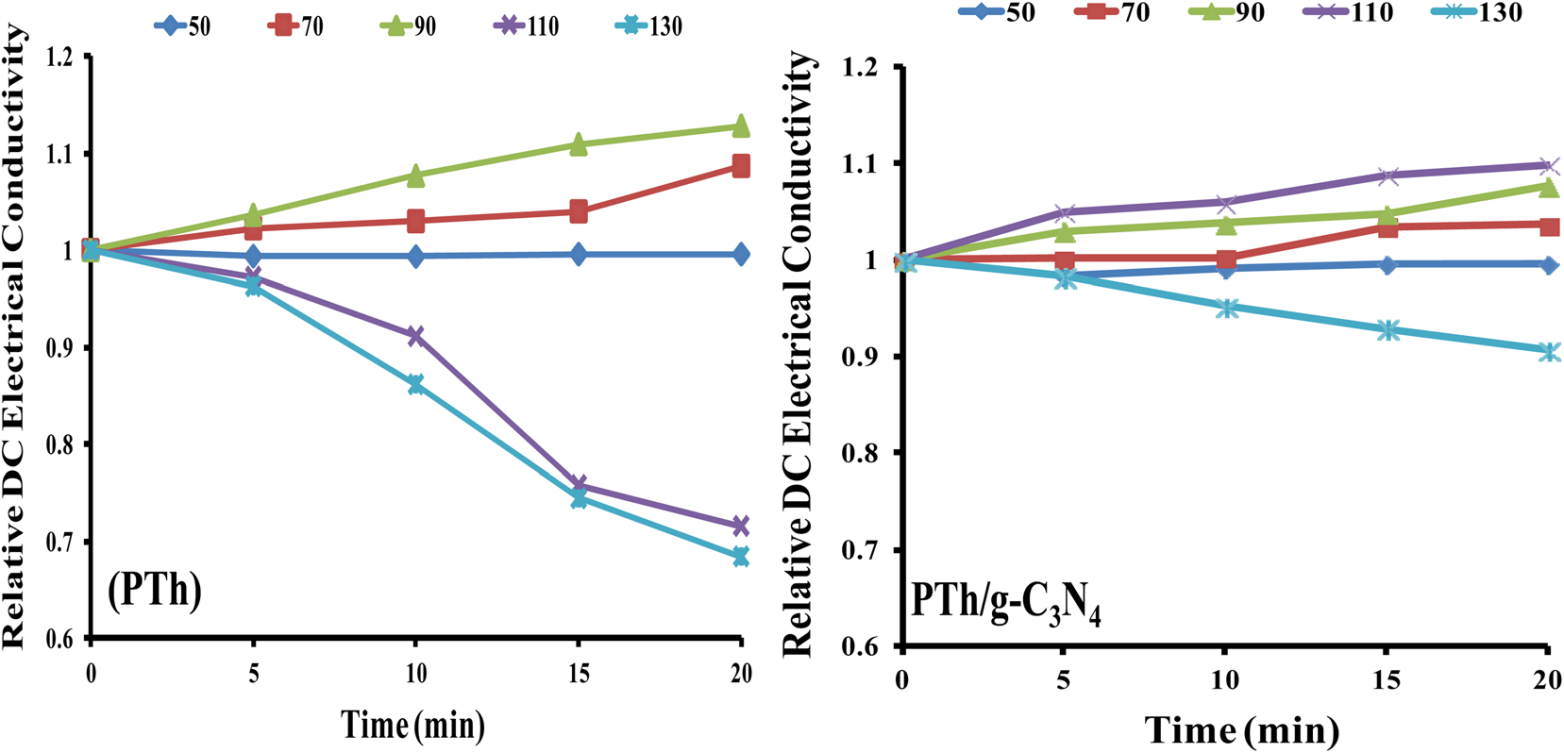
Relative DC electrical conductivity of PTh, and PTh/g-C_3_N_4_ nanocomposite under isothermal ageing environment.

The stability as a function of DC electrical conductivity retaining capacity under isothermal ageing conditions of the nanocomposites PTh and PTh/g-C_3_N_4_ were examined, as shown in Fig. 7.

The equation applied for calculating the relative DC electrical conductivity (*σ*_r,*t*_) at constant temperature was:

2 *σ*_r,*t*_ = *σ*_*t*_/*σ*_0_

in this equation, *σ*_*t*_ and *σ*_0_ is represent the DC electrical conductivities at time ‘*t*’ and ‘0’, respectively.^50–53^

It is clearly noticeable from Fig. 7(a) that PTh exhibited thermally stable electrical conductivity at temperatures 50 °C, 70 °C and 90 °C. At constant temperature up to 90 °C, DC electrical conductivity rises with time indicating the semiconducting nature of PTh. Whereas above 90 °C, there is a continuous fall of the DC electrical conductivity with time, which may be due to the damage of conducting channels as well as the loss of the doping agent in PTh. While for the PTh/g-C_3_N_4_, the DC electrical conductivity was found to be stable at 50 °C, 70 °C, 90 °C and 110 °C and behaves like semiconductor as displayed in Fig. 7(b). The incorporation of g-C_3_N_4_ in PTh stabilises its DC electrical conductivity even in high temperature condition (up to 110 °C) strongly suggesting presence of an electronic interaction between PTh and g-C_3_N_4_, which resists the damaging of its conducting channels and loss of the doping agent. These outcomes displayed that PTh/g-C_3_N_4_ could be an auspicious candidate in various electronic applications even at 110 °C.

### 4.2. Retention of DC electrical conductivity under cyclic ageing condition

**Fig. 8:**
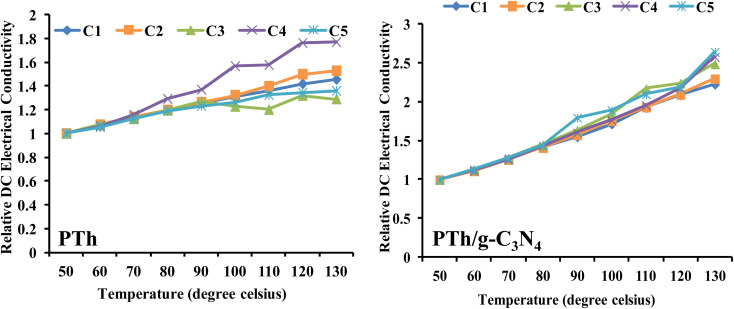
Relative DC electrical conductivity of PTh, and PTh/g-C_3_N_4_ nanocomposite under cyclic ageing environment.

PTh and PTh/g-C_3_N_4_ nanocomposite were also examined for their stability as a function of DC electrical conductivity performance under cyclic ageing conditions, as shown in Fig. 8.

The equation applied for calculating the relative DC electrical conductivity (*σ*_r_) was:

3 *σ*_r =_*σ*_*T*_/*σ*_50_

where: *σ*_*T*_ and *σ*_50_ is representative of the DC electrical conductivities at temperatures *T* and 50 °C.^50–53^

The DC electrical conductivity of both the samples were recorded by four consecutive cycles with continuously rise in temperature up to 130 °C. These findings revealed that DC electrical conductivity increased steadily in each cycle and followed a same pattern in both of the samples (PTh and PTh/g-C_3_N_4_), which may be attributed to the high movability of polarons at elevated temperatures. For PTh {Fig. 8(a)}, in the third and fourth cycles, the conductivity shows different pattern might be as a result of damage of the material and loss of proper conductive channel by repeatedly heating and cooling of PTh. While PTh/g-C_3_N_4_ displayed continuous rise in conductivity with each cycle without losing its conductivity, signifying the presence of electronic interaction between PTh and g-C_3_N_4_.

## 5. Sensing

The electrical conductivity of CPs depends upon many factors *e.g.*, dopants, temperature and fillers *etc.*^17–22,50,51^ In p-type doped conducting polymers, holes like polarons and bipolarons generated act as carriers of charge in the backbone of extended π-conjugated system of these CPs. The mobility of theses polarons and bipolarons charge carriers could considerably be hindered by any electronic interaction with the polymer chain. Adsorption and desorption of any analyte gas on the polymer sensor surface is the basic step in gas sensing procedure to monitor the effective electrical conductivity change of the polymer.^20–25^ Presence of analyte gases can be detected by simple change in electrical conductivity upon adsorption on the sensor's surface, as they interact with polarons of PTh, leading to decline in the DC electrical conductivity. Therefore, here PTh and PTh/g-C_3_N_4_ nanocomposites were tested for change in DC electrical conductivity on simple adsorption and desorption process of the analyte gas.

The % sensing response (*S*) is calculated by the following equation:

4 *S* = (Δ*σ*/*σ*_i_) × 100

where, *σ*_i_ and Δ*σ* are representative of the initial DC electrical conductivity as well as change in DC electrical conductivity during complete exposure of gas respectively.^33,50,51^

### 5.1. Sensing response

**Fig. 9:**
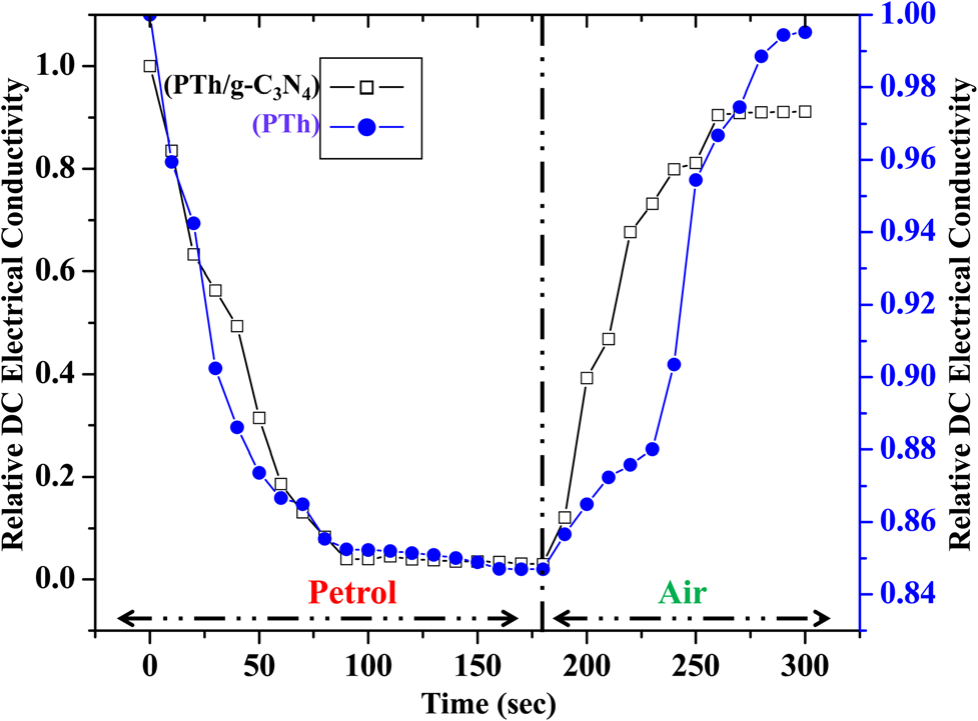
Relative DC electrical conductivity of PTh and PTh/g-C_3_N_4_ nanocomposite in petrol atmosphere.

**Fig. 10:**
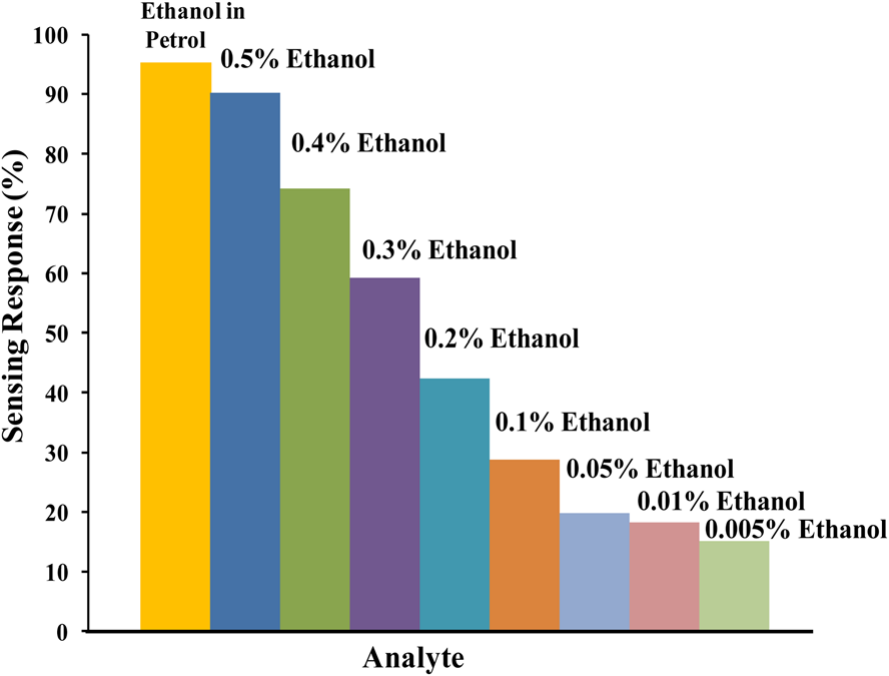
Sensing response of PTh/g-C_3_N_4_ nanocomposite towards different v/v% of ethanol.

Sensing of ethanol in petrol was studied by examining the performance of relative DC electrical conductivity of PTh and PTh/g-C_3_N_4_ nanocomposite upon exposing to petrol vapours followed by ambient air as shown in Fig. 9. The PTh and PTh/g-C_3_N_4_ nanocomposite were finely grinded in powder form and then transformed into pellet form by hydraulic pressure machine. The pellets of each of the sample was attached to the four-in-line probes and placed in a sealed sensing chamber. At first PTh was exposed to petrol vapours followed by room atmosphere for a fixed time. In petrol vapour atmosphere, the DC electrical conductivity of PTh decreased exponentially for about first 90 s and then gets saturated for further change. The loss in electrical conductivity may be due to the ethanol present in petrol because as we know petrol comprises of mainly non-polar hydrocarbons with some mixing of ethanol to increase its octane rating.^33^ The hydrocarbon molecules in petrol being non-polar at ambient temperatures, don't interact electronically with polarons of PTh. The ethanol (C_2_H_5_OH) in petrol interacts with the polarons of PTh, disrupting the conductivity channels by impeding the movement of some polarons of PTh, causing decline in DC electrical conductivity. As PTh pellet exposed to ambient air, the relative DC electrical conductivity began to increase and came back to its initial value within 95 s and then flattened may be as a reason of complete desorption of ethanol from the PTh surface. Similarly for the PTh/g-C_3_N_4_ nanocomposite, the relative DC electrical conductivity declined by exposing the pellet of PTh/g-C_3_N_4_ nanocomposite to petrol and reverted back to its initial value within 80 s in air. The sensing response of PTh and PTh/g-C_3_N_4_ nanocomposite were found to be 15.55% and 94.73% respectively. The sensing response calculated in PTh/g-C_3_N_4_ nanocomposite was 6.1 times higher than that of PTh may be due to the high adsorption–desorption on PTh/g-C_3_N_4_ nanocomposite surface. Thus, incorporation of g-C_3_N_4_ in PTh, creating high surface area with proper advancing in conducting channels of PTh for adsorption and desorption of analyte gases. The sensing response of PTh/g-C_3_N_4_ toward ethanol in petrol as well as at different volume percentage (0.5%, 0.4%, 0.3%, 0.2%, 0.1% and 0.05%) of ethanol in *n*-hexane were also determined that is 95.3345%, 90.152%, 74.242%, 59.091%, 42.424%, 28.788%, 19.697%, 18.188% and 15.152% respectively as shown in Fig. 10. The sensing response towards petrol was the highest among them may due to the highest ethanol mixing in petrol. As the volume percentage of ethanol decreases, the sensing response decreases due to fewer interactions of lone pair of ethanol with polarons of PTh. As a result, one can say that considerable variation in conductivity was seen at high concentrations.

### 5.2. Ethanol sensing

**Fig. 11:**
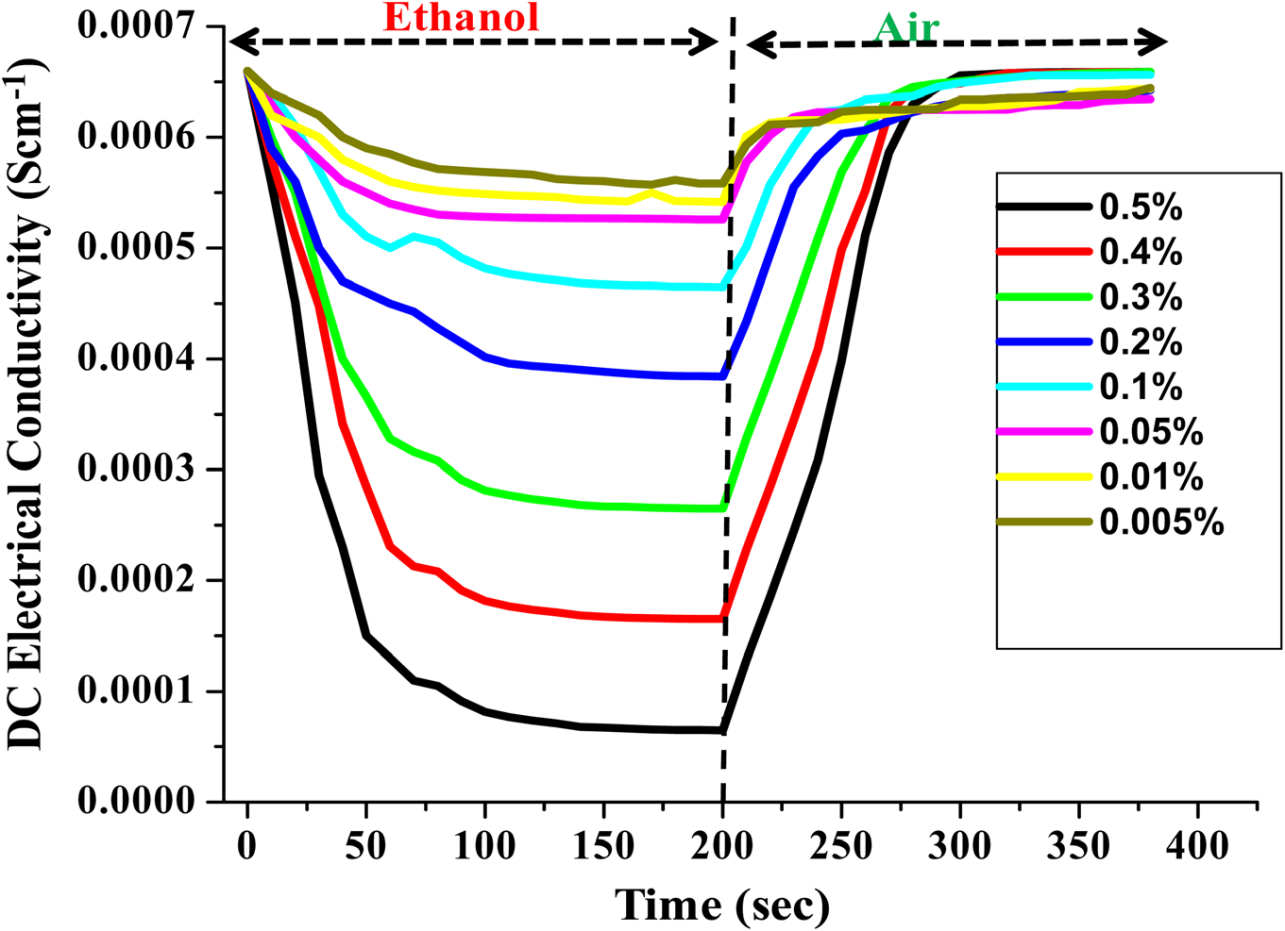
Steady-state response of DC electrical conductivity of PTh/g-C_3_N_4_ nanocomposite in different v/v% of ethanol atmosphere.

In order to justify the PTh/g-C_3_N_4_ nanocomposite's sensing response towards ethanol in petrol, the DC electrical conductivity performance of PTh/g-C_3_N_4_ nanocomposite was also recorded for different volume percentage of ethanol in *n*-hexane as shown in Fig. 11. The DC electrical conductivity follows the same pattern as it is for petrol. As the volume percentage of ethanol increases, the variation in DC electrical conductivity of PTh/g-C_3_N_4_ nanocomposite increases, may be due to high interaction of ethanol with the polarons of PTh/g-C_3_N_4_ nanocomposite and gets saturated after few seconds. While in ambient air, conductivity completely reverted to its initial value by simple desorption of ethanol from PTh/g-C_3_N_4_ nanocomposite surface.

### 5.3. Reversibility test

**Fig. 12:**
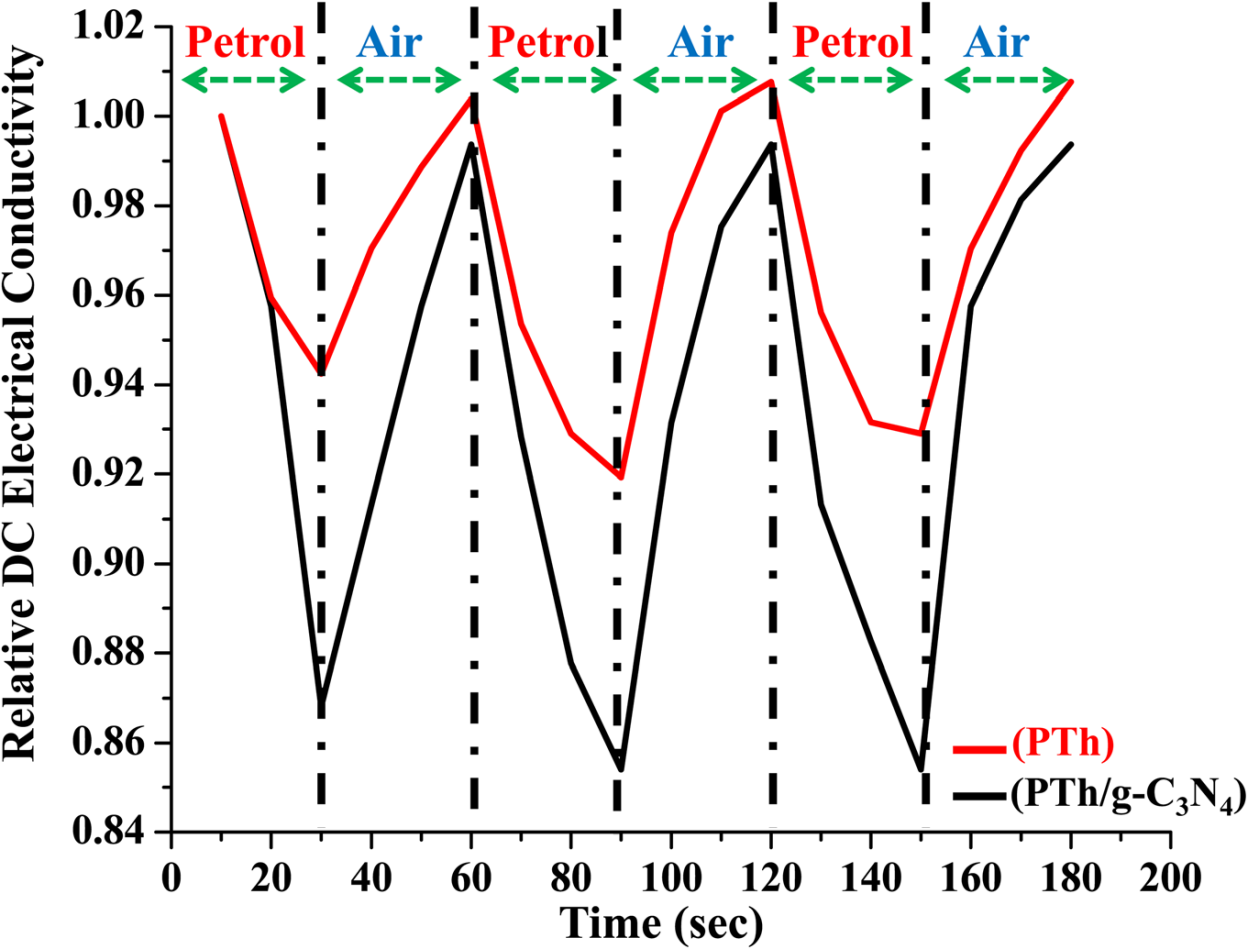
Relative DC electrical conductivity of PTh and PTh/g-C_3_N_4_ nanocomposite on alternate exposure to petrol and ambient air with respect to time.

The PTh and PTh/g-C_3_N_4_ nanocomposite were tested for their rapid adsorption and desorption of analyte by observing the variation in relative DC electrical conductivity in environment of petrol for 30 s followed by ambient air for the next 30 s consecutively for three 60 s cycles as shown in Fig. 12. Both of the samples showed excellent reversibility with minimum loss in DC electrical conductivity in repeated three consecutive cycles. In PTh/g-C_3_N_4_ nanocomposite the variation in relative DC electrical conductivity was more superior to that of PTh, which may be due to more adsorption–desorption of analyte on PTh/g-C_3_N_4_ nanocomposite surface than that of PTh.

### 5.4. Selectivity

**Fig. 13:**
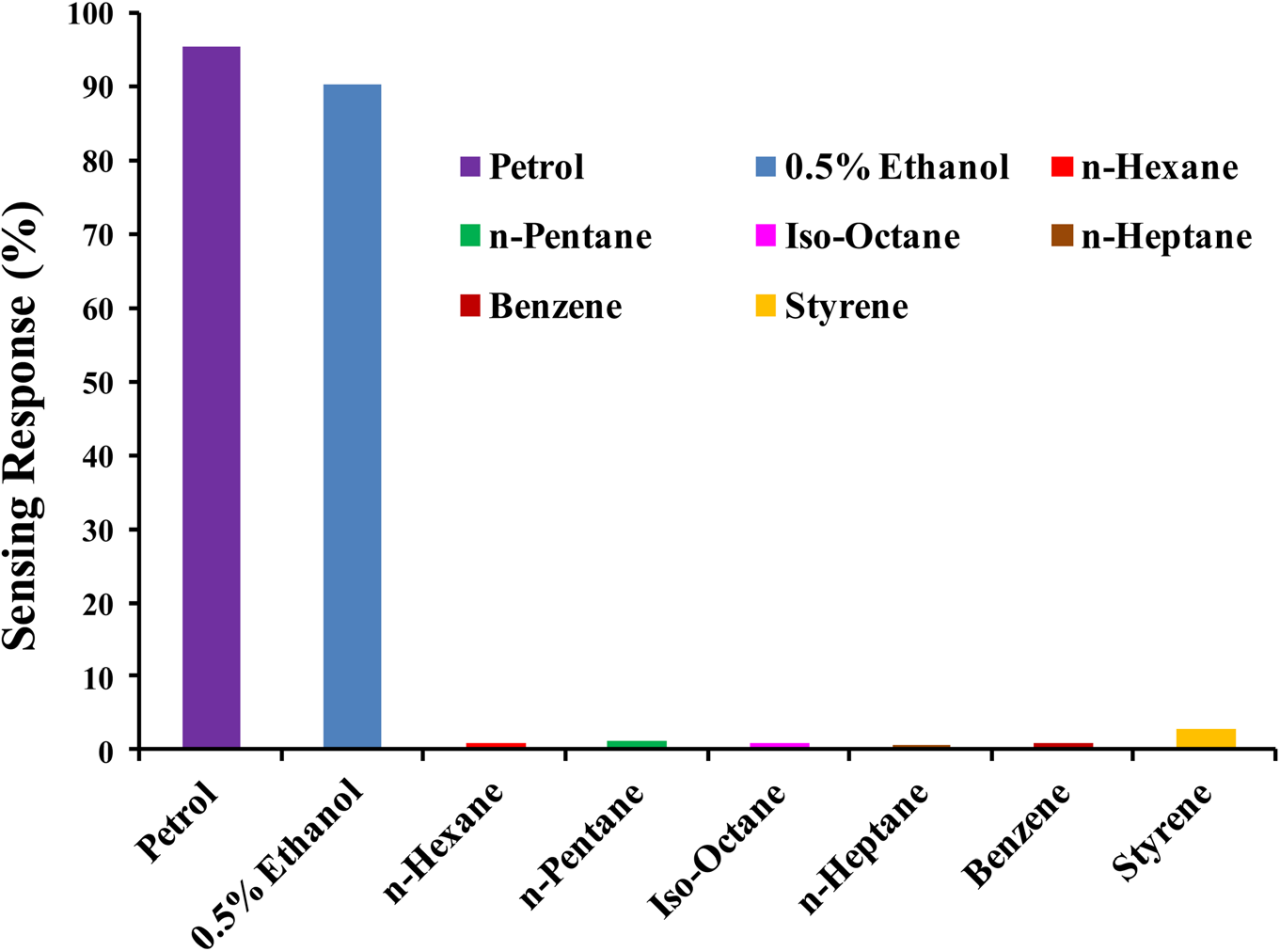
Selectivity of PTh/g-C_3_N_4_ nanocomposite towards ethanol *vs.* different hydrocarbons.

High selectivity is an essential criterion for a gas sensor in order to qualify as reliable and applicable. The PTh/g-C_3_N_4_ nanocomposite sensing response toward ethanol mixing in petrol, 0.5% ethanol in *n*-hexane, *n*-hexane, *n*-pentane, iso-octane, *n*-heptane, benzene, styrene at room temperature (27 °C) are displayed in Fig. 13. High selective response of PTh/g-C_3_N_4_ nanocomposite toward ethanol may be due its availability of lone pairs of oxygen which interacts with polarons of PTh, causing significant loss of DC electrical conductivity (high sensing response). While for the other analytes (hydrocarbons) tested, the sensing responses were negligible in comparison of ethanol may be of their non-polar nature. Thus the lower the polarity/free lone pairs of electrons in the analyte, lower the conductivity change occurred in PTh/g-C_3_N_4_ nanocomposite, makes ethanol highly selective in comparison of different hydrocarbon compounds.

### 5.5. Sensing mechanism

**Scheme 1:**
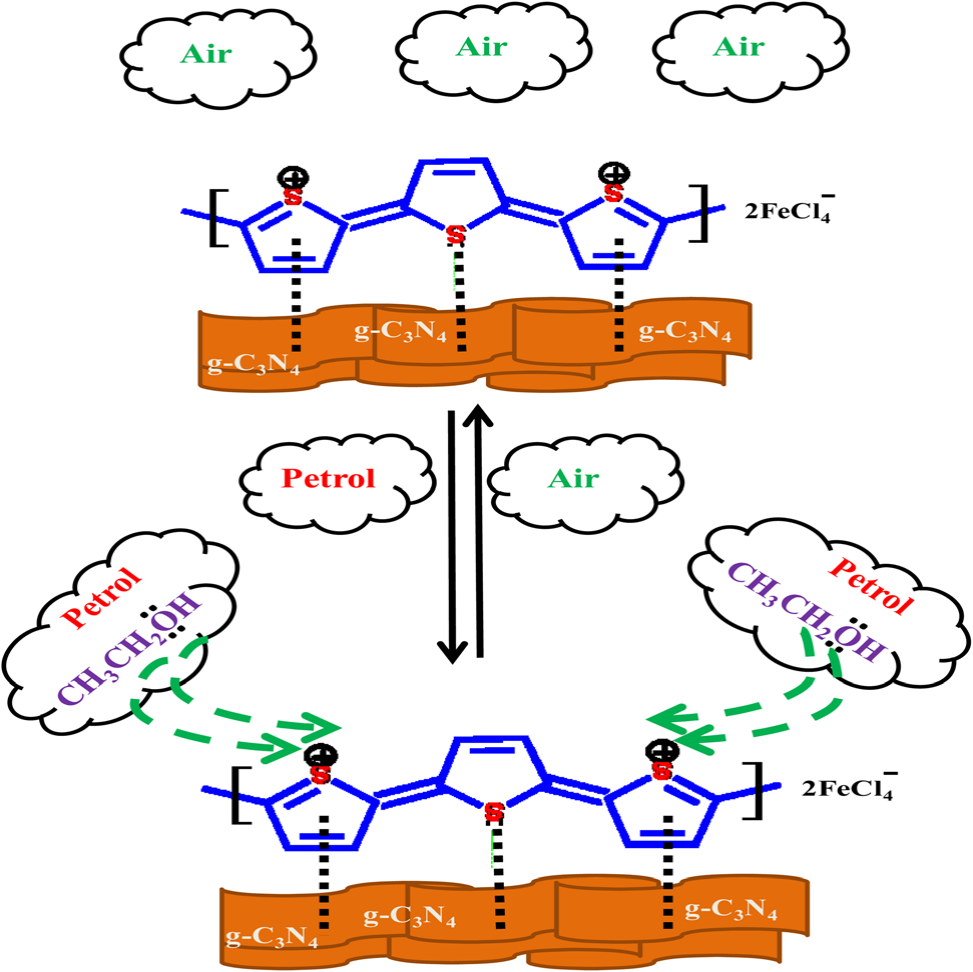
Proposed mechanism of interaction of ethanol with the PTh/g-C_3_N_4_ nanocomposite.

Sensing mechanism of PTh/g-C_3_N_4_ nanocomposite is explained by the DC electrical conductivity behaviour on simple adsorption and desorption mechanism of analyte at room temperature, as depicted in Scheme 1. In PTh/g-C_3_N_4_ nanocomposite, graphitic-carbon nitride get interacted with pi-bonds of thiophene rings in polythiophene and form a nanocomposite providing high surfaced platform for sensing of analytes. It is already known that petrol is comprises of majority of hydrocarbons and some ethanol mixing to raise its octane number. The hydrocarbon molecules in petrol being nonpolar at ambient temperature, do not interact with the polarons of polythiophene in PTh/g-C_3_N_4_ nanocomposite. Ethanol is predicted as the only candidate that could be accountable for the variation in the conductivity of PTh/g-C_3_N_4_ nanocomposite upon exposure to petrol vapors. In presence of petrol atmosphere, the lone pairs of ethanol interact with the polarons of PTh/g-C_3_N_4_ nanocomposite, which in turn hindered the movement of polarons, eventually declination of DC electrical conductivity with time. While in ambient air condition, the ethanol molecules and petrol gets desorbed from the sensor surface, resulting in reverting its conductivity to it's about initial values, indicating complete desorption of ethanol molecules mixed in petrol. Thus, the above mechanism indicates that polarons mobility is controlled by the simple adsorption and desorption of ethanol on PTh/g-C_3_N_4_ nanocomposite surface.

## 6. Conclusions

In this paper, we synthesized PTh and PTh/g-C_3_N_4_ nanocomposite, characterized and studied for their stability of DC electrical conductivity and sensing response toward ethanol mixing in petrol. The stable nature of DC electrical conductivity of PTh/g-C_3_N_4_ nanocomposite under accelerated isothermal and cyclic ageing condition were demonstrated and found significantly higher than that of PTh. Also the PTh/g-C_3_N_4_ nanocomposite showed rapid response and high reversibility towards ethanol mixing in petrol and ethanol prepared in *n*-hexane with a detection limit of 0.005%. The sensing response in PTh/g-C_3_N_4_ nanocomposite towards ethanol mixing in petrol was 6.1 times higher than that of PTh at room temperature. In addition of excellent sensing response, this research found that PTh/g-C_3_N_4_ nanocomposite might be used as a semiconducting material in a variety of electronic and electrical devices at high temperature conditions.

## Conflicts of interest

The authors declare that they have no known competing financial interests or personal relationships that could have appeared to influence the work reported in this paper.

## Supplementary Material
